# ssREAD: A Single-cell and Spatial RNA-seq Database for Alzheimer’s Disease

**DOI:** 10.1101/2023.09.08.556944

**Published:** 2023-09-12

**Authors:** Cankun Wang, Megan McNutt, Anjun Ma, Hongjun Fu, Qin Ma

**Affiliations:** 1 Department of Biomedical Informatics, The Ohio State University, OH 43210, USA; 2 Department of Neuroscience, The Ohio State University, OH 43210, USA

**Keywords:** Alzheimer’s Disease, Web database, single-cell and single-nucleus RNA sequencing, Spatial transcriptomics

## Abstract

Alzheimer’s Disease (AD) is a neurodegenerative malady predominantly affecting the elderly and exhibits its debilitating effects on a dementia-prone population. Recently, the advent of innovative technologies, such as single-cell and single-nucleus RNA-sequencing (scRNA-seq & snRNA-seq) and spatial transcriptomics (ST), has reformed our investigative approaches toward comprehending AD’s neuropathological intricacies and underpinning regulatory mechanisms, encompassing sub-cellular, cellular, and spatial dimensions. In light of the overwhelming proliferation of single-cell and ST data associated with AD, the imperative for a comprehensive, user-friendly database that addresses the scientific community’s analytical demands has never been more paramount. Introduced initially in 2020, scREAD presented itself as a pioneering repository that systematized publicly available scRNA-seq and snRNA-seq datasets derived from post-mortem human brain tissues and mouse models mirroring AD pathology. Here, we introduce ssREAD, a substantial upgrade over scREAD, enriching the platform with a broader spectrum of datasets, an optimized analytical pipeline, and enhanced usability and visibility. Specifically, ssREAD amalgamates an impressive portfolio of over 189 datasets extracted from 35 distinct AD-related scRNA-seq and snRNA-seq studies, encompassing a staggering 2,572,355 cells. In addition, we have diligently curated and archived 300 ST datasets, originating from 12 human and mouse brain studies, which include two focused on AD and ten control studies. Every dataset within our repository is meticulously annotated, bearing critical identifiers including species, gender, brain region, disease/control status, age, and AD stages. Besides the collection of above datasets in ssREAD, it delivers an exhaustive analysis suite offering cell clustering and annotation, inference of differentially expressed and spatially variable genes, identification of cell-type-specific marker genes and regulons, and spot deconvolution for integrative analysis of ST and scRNA-seq & snRNA-seq data from public domains. All these resources are freely accessible through a user-friendly, consolidated web portal available at https://bmblx.bmi.osumc.edu/ssread/.

## Introduction

Alzheimer’s disease (AD) is a progressive neurodegenerative disorder, the most common form of dementia worldwide. Over 57 million individuals globally suffer from this debilitating condition^1^. Despite the significant strides in medical research and development, therapeutic interventions for AD remain distressingly ineffective. This glaring lacuna in clinical therapeutics underscores the urgency to probe into the intricate molecular mechanisms underlying the disease’s cellular and regional susceptibility that are still largely enigmatic^2^. Recent high-throughput sequencing technologies, particularly single-cell RNA-sequencing (scRNA-seq) and single-nucleus RNA-sequencing (snRNA-seq) have cast fresh light on our exploration of AD pathogenesis. To study the cellular heterogeneity of the brain and reveal the complex cellular changes in AD, we launched scREAD in 2020^3^. By then, it was the first database dedicated to managing public AD-related sc/snRNA-Seq data from human and mouse brain tissue. As the sequencing technology and scientific inquiry rapidly evolved, more studies are discerning the spatial information of differentially expressed genes (DEGs) associated with AD pathology, the interconnectedness of DEGs related to AD biomarkers, DEGs enriched in specific cell subtypes, cell-cell communications, and regional and cellular vulnerability in AD^4^. Spatial transcriptomics (ST) revolutionized our understanding of neurobiology and AD pathogenesis by enabling the visualization of gene expression patterns within their spatial context. Yet, these public-available scRNA-seq, snRNA-seq, and ST data have not been well collected and managed by any AD databases. In addition, the remarkable potential usage of these datasets is accompanied by the formidable challenge of data aggregation, analysis, and interpretation, necessitating substantial computational resources and bioinformatics expertise.

To address these burgeoning complexities and to meet the scientific community’s growing demand for comprehensive, integrated, and accessible data analysis, we introduce **ssREAD** (Single-cell and Spatial RNA-seq databasE for Alzheimer’s Disease). It encapsulates 300 ST and 189 sc/snRNA-seq AD-related data. These sequencing data enable researchers to investigate transcriptomic alterations in AD compared to the control and their regulatory mechanisms at various resolutions: sub-cellular, cellular, and spatial levels^5,6^, which will help uncover the pathogenesis of AD. We also highlight the sc/snRNA-seq and ST data analysis framework in ssREAD, including cell clustering and annotation, differentially expressed and spatially variable gene identification, cell-type-specific regulon inference, and functional enrichment analysis. Moreover, the integrative exploration of ST and sc/snRNA-seq data revealed nuanced molecular landscapes that underlie AD, emphasizing ssREAD’s unparalleled capability. Beyond that, ssREAD also delivers marked improvements to the user interface. These modifications, grounded in user-centric design principles, advance visibility and usability, fostering an environment conducive to intuitive data visualization and streamlined querying.

## Results

### 1. Overview of ssREAD

ssREAD comprises 300 ST datasets from 12 AD-related studies and 189 scRNA-seq and snRNA-seq datasets from 35 studies. All datasets are collected and downloaded from Broad Insitute SingleCellPortal, Gene Expression Omnibus (GEO), and Synapse^7^ (**Supplementary Figure S1**). ssREAD has a considerable 159% increase in the number of sc/snRNA-seq datasets than the previous scREAD (from 73 to 189), encompassing over three times the total cell and nucleus count.

**Figure 1.**
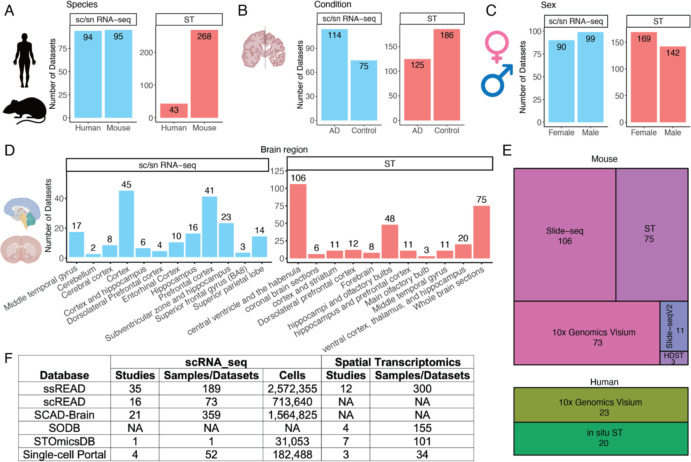
ssREAD Data characteristics and statistics (as of May 2023). (A-D) Barplots show the number of datasets by species, sex, condition, and brain regions, respectively. (E) Treemap presents the breakdown of technologies deployed in mouse and human studies. (F) The comparative table showcases the number of studies and datasets across extant AD databases and data collection sources.

In our pursuit of thoroughness, each dataset is meticulously annotated, providing pertinent details such as species, gender, brain region, disease/control distinction, and AD stages. In the realm of species, 94 out of 189 sc/snRNA-seq datasets are from human samples, whereas 95 datasets are mouse. In contrast, mouse datasets are prominently represented with 268 ST datasets, four times more than human ST datasets (Figure 1A). When demarcating datasets based on the AD condition, 114 sc/snRNA-seq datasets are assigned to the AD group, as opposed to the 75 datasets associated with control scenarios (Figure 1B). This distribution takes a nuanced shift within ST samples, with AD-oriented datasets numbering 125 and control datasets as 185. Sex-specific stratification is also evident within the ssREAD construct. Male representation encapsulates 99 datasets from sc/snRNA-seq and an additional 159 datasets sourced from ST methodologies (Figure 1C), compared to 90 sc/snRNA-seq and 142 ST datasets for females. Moreover, delineation across brain regions further accentuates the depth of ssREAD, with distinct datasets marked as per techniques, either sc/snRNA-seq or ST (Figure 1D). Technological diversity, a cornerstone for generating a multifaceted spatial transcriptomics landscape, is manifestly displayed within ssREAD (Figure 1E). For the mouse model, technologies span from Slide-seq, with 106 samples, to HDST, which features across three samples. The human segment is apparently inclined towards 10x Visium, accounting for 23 samples, closely trailed by In-situ ST, represented with 20 samples.

Benchmarking ssREAD against contemporaneous repositories underscores its unparalleled stature. Compared with some existing databases, such as scREAD^3^, SCAD-Brain^8^, SODB^9^, STOmicsDB^10^, and Single-Cell Portal, ssREAD has the most decadent AD-related samples collected for both sc/snRNA-seq and ST data with great depth and breadth (Figure 1F). Overall, ssREAD leads to a comprehensive collection of AD-related scRNA-seq, snRNA-seq, and ST datasets, offering both breadth and depth of data that stand unrivaled compared to other available resources or databases.

**Figure 2.**
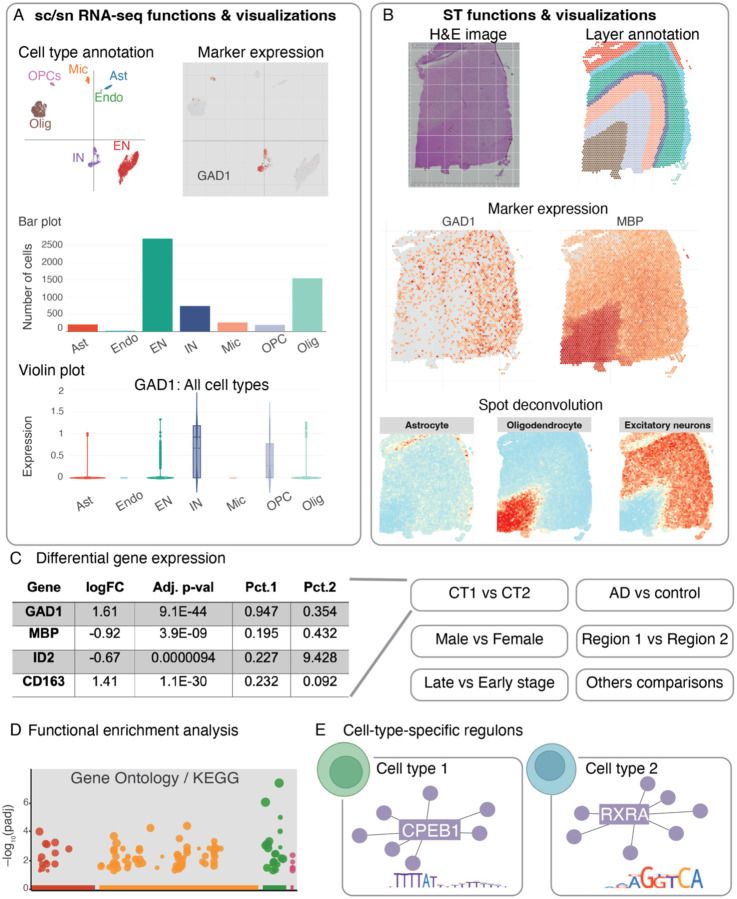
Overview of ssREAD functions. (A) Features and visual representations related to sc/snRNA-seq, encompassing cell type annotations, marker gene expressions, and graphic depictions via bar and violin plots. (B) Functions and visualizations pertinent to ST, highlighting H&E imagery, layer annotations, marker gene expressions, and spot deconvolutions. (C) DEG analysis, with comparisons drawn between categories like Cell Type 1 vs. Cell Type 2 (CT1 vs. CT2), AD vs. Control, Male vs. Female, Brain Regions 1 vs. 2, etc. (D) Functional enrichment analysis focusing on GO ontology and KEGG pathways. (E) Predictions of cell type-specific regulons. The following abbreviations are used for cell types: Astrocytes (Ast), Endothelial Cells (Endo), Excitatory Neurons (EN), Inhibitory Neurons (IN), Microglia (Mic), Oligodendrocytes (Olig), and Oligodendrocyte Precursor Cells (OPC).

Our ssREAD collects the above data and offers comprehensive, in-depth data analyses and result interpretations. For sc/snRNA-seq data, ssREAD provides functions including cell clustering, cell type annotation, marker gene expression visualization, and cell proportion analysis (Figure 2A and **Supplementary Table S2**). For ST data, it provides visualizations for original spatial H&E image, layer/tissue architecture/spatial domain annotation, marker gene expression on spatial map, and spot deconvolution (Figure 2B). Differentially expressed genes (DEGs) can be identified for cell types in sc/snRNA-seq data or spatial layers in ST data (Figure 2C). Cross-data queries and analyses are also facilitated, including comparative studies between male and female subjects across various datasets. Additionally, spatially variable genes (SVGs) can be identified via spaGCN^11^ from ST data to show marker genes with spatially resolved expression patterns that may be related to tissue functions. Functional enrichment analysis is included to identify pathways or gene ontology enriched by DEGs or SVGs (Figure 2D). Moreover, ssREAD also features cell-type-specific (or layer-specific) regulons for individual datasets and the integrated cell atlas (Figure 2E), focusing on cellular and regional vulnerability in AD.

To ensure ease of use, all analytical results are displayed via a user-friendly and single-access web portal that frees AD researchers from the requirement of extensive programming knowledge. We offer new interactive plots for visualizing cells and spatial spots, including scatter plots, bar plots, and violin plots, as well as real-time analyses for DEGs, SVGs, and functional enrichment queries. Furthermore, all datasets, including author-provided metadata and cell type labels, are provided in ready-to-analyze formats (e.g., .h5ad and .h5seurat), compatible with analysis tools such as Seurat and Squidpy for further analysis.

### 2. Spatially-informed Subpopulation Analysis Reveals Cellular Heterogeneity in AD

**Figure 3.**
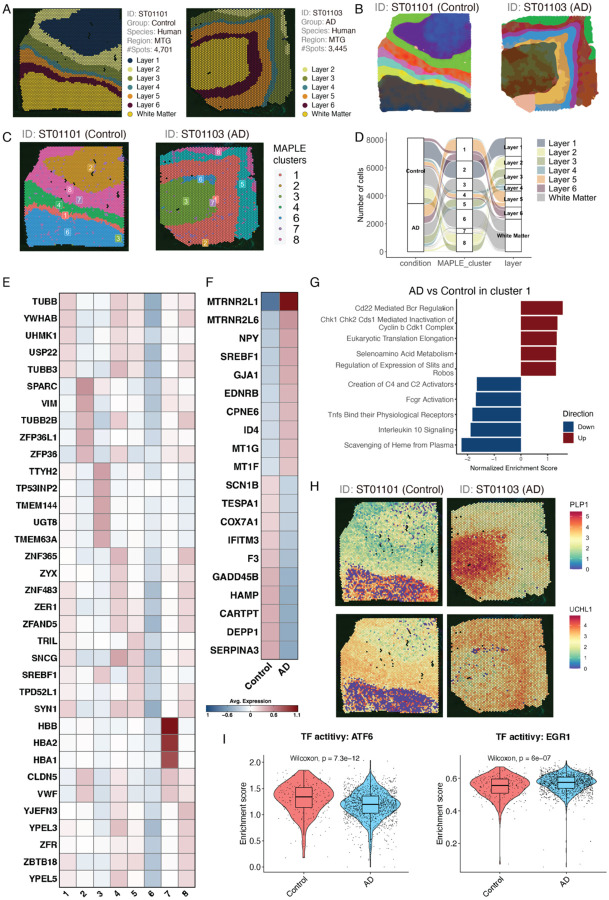
Multi-dimensional Analysis of Spatially-informed Sub-populations. (A) Annotation of the six cortical layers alongside the adjacent white matter within two human middle temporal gyrus (MTG) brain samples (ST01101 and ST01103). (B) Detection of spatial domains by RESEPT. (C) Visualization using MAPLE to elucidate shared or unique spatial domains identified across the two Spatial Transcriptomics samples (ST01101 and ST01103). (D) Alluvial diagrams showcasing the progression of cells: originating from individual samples, aggregating into joint subpopulations, and culminating in layer annotations. (E) A heatmap depicting genes specific to the MAPLE-derived clusters for both AD and Control samples. (F) Heatmap representing the top 10 upregulated and top 10 downregulated genes distinguishing AD from Control within Cluster 1. (G) Gene Set Enrichment Analysis (GSEA) of differentially expressed genes from (E) plotted against REACTOME pathways. The bar plot shows the top 10 upregulated and downregulated pathways, accompanied by normalized enrichment scores. (H) Spatial feature plots highlighting the variance in gene expression of PLP1 and UCHL1 from Cluster 1, segregated by AD and Control samples. (I) Violin plots showcasing the activity of two selected TFs between AD and Control, with associated p-values derived from a Wilcoxon rank-sum test.

To illustrate the ST data analysis workflow and functions, we used two ST data (ST01101 and ST01103), labeled by six cortical layers and the adjacent white matter in two human middle temporal gyrus (MTG) brain samples based on the information provided in the original study^12^ (Figure 3A). ssREAD implements RESEPT^13^, a deep-learning framework for spatial domain detection, to provide a precise delineation of the tissue architecture and functional zones in both control and AD brain tissues (Figure 3B). Moreover, the potential of ssREAD in navigating the complex spatial information of AD was further exemplified through a multi-dimensional exploration of spatially informed sub-populations via MAPLE^14^ (Figure 3C). Noted that MAPLE has a critical multi-sample design considering information sharing across samples and accommodating spatial correlations in gene expression patterns within samples. Thus, clusters MAPLE identifies are sample-specific and could be either shared among samples (e.g., cluster 1 in both ST01101 and ST01103) or unique in individual samples (e.g., cluster 5 only in ST01103). This analysis demonstrated ssREAD’s ability to untangle the spatial complexity inherent in the transcriptomic landscape of AD. To further illuminate these cellular dynamics, we mapped the MAPLE cluster annotations in individual samples to their corresponding layer annotations (Figure 3D).

At the molecular level, we identified DEGs in each MAPLE cluster (Figure 3E) and between control and AD samples within individual MAPLE clusters (Figure 3F). The expression pattern of these DEGs underscores the molecular heterogeneity in MAPLE clusters (subpopulations) and between AD and the control (Figure 3F). For example, the expression of DEPP1, also known as PGC-1α (peroxisome proliferator-activated receptor gamma coactivator 1-alpha), was found to be significantly lower in AD than the control in our dataset. The PGC-1α is highly responsive to numerous forms of environmental stress, including temperature and nutritional status. Several studies have reported that the level of PGC-1α significantly decreases in the brains with AD compared to control brains^15–18^. PGC-1α has thus been suggested to contribute to the improvement of AD pathophysiology. Additionally, the pathway enrichment analysis on AD and control DEGs in cluster 1 showed both upregulated and downregulated pathways. Interestingly, all five downregulated pathways are associated with immune responses/functions (Figure 3G), which may highlight the important role of microglia and other immune cells in AD pathogenesis^19–22^. Besides DEG and DEG enriched pathways, we also identified 305 SVGs in cluster 5, such as PLP1 and UCHL1 (Figure 3H), which show clear spatial expression patterns that are linked to specific tissue layers. Interestingly, both genes have also been found to be associated with AD^23,24^.

To further investigate the regulatory mechanisms in different clusters between the above Control (ST01101) and AD (ST01103) samples, we implement DeepMAPS^25^ in the ssREAD framework for spatial transcriptomics-guided gene regulatory network analysis. This facilitated a comprehensive investigation into the interconnected relationships among key transcription factors (TFs) and their associated genes. Our analysis highlighted a network comprising 10 TFs (i.e., NR1H3, SREBF1, ATF6, TAL1, SOX10, NFYA, AR, MYC, HIF1A, and EGR1) and 2,164 genes regulated by those TFs (**Supplementary Table S3**). This network, interconnected with the enriched genes from gene modules identified by DeepMAPS, underscores the dynamic and intricate interactions between critical TFs and their downstream regulated genes. Further dissection of these TFs showed differences in TF activities between control and AD samples, for example, ATF6 and EGR1 (Figure 3I). These two regulators are reported to be associated with AD pathology. ATF6, a key player in unfolded protein response to endoplasmic reticulum (ER) stress, has been found to reduce amyloid-beta (Aβ) toxicity via the downregulation of β-site APP-cleaving enzyme 1 (BACE1)^26^. On the other hand, EGR1 may play a role in keeping the brain’s cholinergic function intact in the preclinical stages of AD via the upregulation of acetylcholinesterase (AChE)^27^, and EGR1 regulates tau phosphorylation and Aβ synthesis in the brain by controlling activities of Cdk5 and BACE-1, respectively^28^. Overall, our data-driven analysis of ST data provides a stepping stone for future studies aimed at deciphering the complex molecular pathogenesis and novel therapeutic targets.

### 3. ssREAD Unveils AD Pathophysiology through an Integrated Analysis of Spatial and Single-cell Transcriptomics

**Figure 4.**
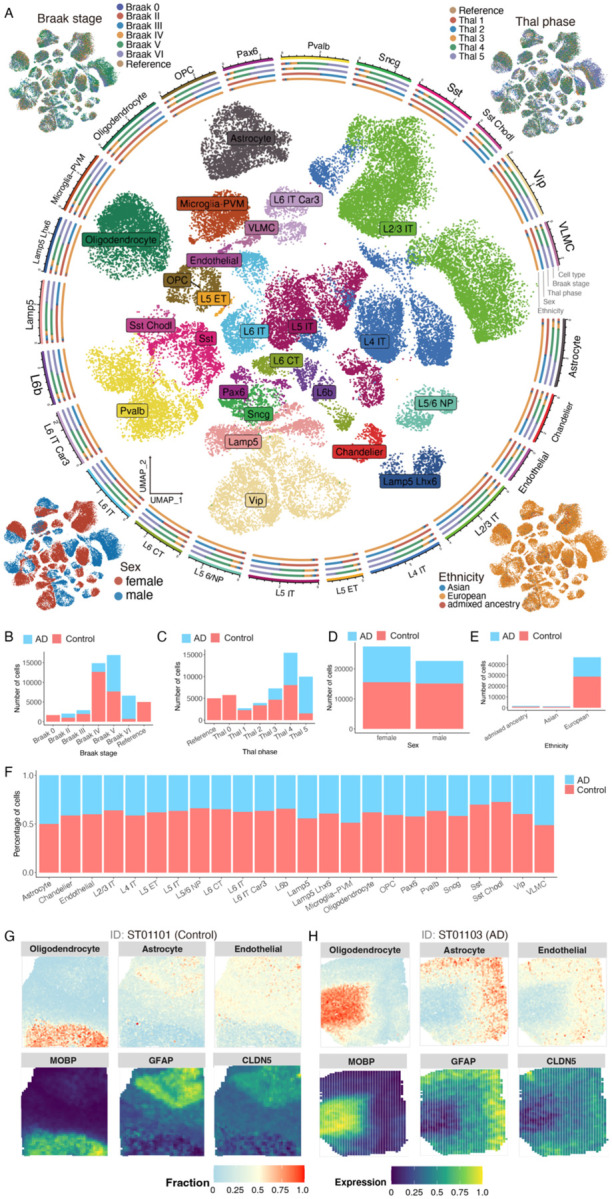
ssREAD Facilitates Comprehensive Spatial Transcriptomics Analyses Synergized with scRNA-seq Datasets. (A) UMAP representation of cell types derived from the Seattle Alzheimer’s Disease Brain Cell Atlas (SEA-AD). Clockwise from the top left, corner insets elucidate the Braak stage, Thal phase, ethnicity, and sex attributes. (B-E) Barplots visualizing the distribution of cells based on the Braak stage, Thal phase, sex, and ethnicity, categorized by the condition in the atlas. (F) Barplots displaying the fractional representation of cell types, contrasting AD and control within the SEA-AD atlas. (G-H) Deconvolution analysis of cell types between ST samples (ST01101 and ST01103) and the scRNA-seq SEA-AD atlas, presenting the cell type fractions for oligodendrocytes, astrocytes, and endothelial cells. The cell type fractions are positioned beneath their corresponding marker gene expression indicators: MOBP for oligodendrocytes, GFAP for astrocytes, and CLDN5 for endothelial cells, with analyses segregated into control (G) and AD (H) conditions.

One of the most prominent analyses that ssREAD can power is the spot deconvolution enabled by the integration of sc/snRNA-seq and ST data. A cornerstone in ssREAD is the utilization of the Seattle Alzheimer’s Disease Brain Cell Atlas (SEA-AD), an atlas that includes cells derived from the health and AD human middle temporal gyrus (MTG)^29^. The SEA-AD atlas originally encompassed an extensive dataset of 378,211 cells. We employed the sketch-based analysis feature in Seurat v5 to streamline our analysis, which strategically selects a subset or ‘sketch’ of 50,000 cells. This analytical decision was driven by achieving computational efficiency while ensuring a robust representation of cellular diversity. Such an atlas painted a detailed cellular tableau, effectively revealing cell distributions of 23 cell types (Figure 4A). Beyond cell types, the atlas also includes a nuanced statistical breakdown of the cells, distinctly categorized by the Braak stage, Thal phase, gender, and ethnicity, offering a comprehensive glimpse into the atlas’s cellular constitution (Figure 4B–F). As shown, there is no batch effect among samples regarding Braak stage, Thal phase, and ethnicity. However, there are obvious sex-oriented differences in cell clusters, which may contribute to the possible pathological sex-bias differences in AD.

Bridging the understanding between single-cell and spatial data, ssREAD was instrumental in performing a cell-type deconvolution analysis between the ST datasets and the SEA-AD atlas using the CARD R package^30^. This synergetic approach underscored the presence and dynamics of critical cell types, including oligodendrocytes, astrocytes, and endothelial cells, across both the control (ST01101) and AD (ST01103) datasets. The cellular insight was further illuminated by visualizing the expression of marker genes, such as MOBP for oligodendrocytes, GFAP for astrocytes, and CLDN5 for endothelial cells, distinguishing between control (Figure 4G) and AD conditions (Figure 4H). The seamless fusion of snRNA-seq and spatial transcriptomics data, as facilitated by ssREAD, positions this integrated methodology as a potent tool for in-depth cellular analysis, demonstrating the platform’s pivotal role in multi-dimensional data integration and offering transformative insights into AD. As a result, we showcase the capability of ssREAD in integrating sc/snRNA-seq and ST data with computational tools like MAPLE, shedding light on the multi-dimensional dynamics of spatially informed subpopulations in AD. By unveiling the biological pathways and regulatory networks associated with disease progression, ssREAD allows a comprehensive understanding of the cellular and molecular landscape in AD, thus bringing us a step closer to unraveling the mysteries of this complex neurodegenerative disease.

### 4. Unveiling Sex-Specific Differences in Alzheimer’s Disease at the Cellular Level

**Figure 5.**
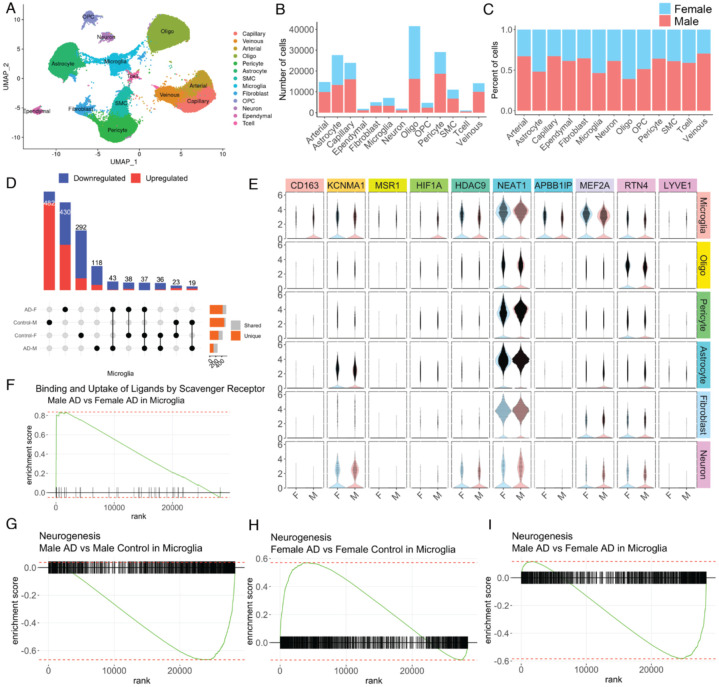
Exploration of Sex-Specific Differences at the Cellular Level in AD (A) UMAP visualization of the single-cell data used in the analysis, with different cell types color-coded. (B-C) Bar plots illustrate the count and proportion of each cell type, segregated by sex. This reveals any potential differences in cellular composition between male and female samples. (D) UpSet plot showing the unique and shared DEGs across four groups: Male AD patients, Female AD patients, Male controls, and Female controls. (E) Violin plots for a selection of 10 genes, illustrating their expression levels in males versus females, and across various cell types (microglia, oligodendrocytes, pericytes, astrocytes, fibroblasts, and neurons). (F) Gene Set Enrichment Analysis (GSEA) plot showing the enrichment of genes involved in binding and uptake of ligands by scavenger receptors. (G-H-I) GSEA plots showing the enrichment of genes involved in neurogenesis for three different comparisons: Male AD vs. Male Control in Microglia (G), Female AD vs. Female Control in Microglia (H), and Male AD vs. Female AD in Microglia (I). These plots highlight the sex-specific differences in neurogenesis-related gene activity under AD conditions.

Besides the data-driven analysis that can be elaborated from ssREAD, we also showcase the ability to generate new biological hypotheses that can be powered by the database. Capitalizing on the wealth of information offered by sc/snRNA-seq data, our investigation delves into the intricate, sex-specific nuances of AD at the cellular level. Using ssREAD, we were able to elucidate the AD heterogeneity between male and female, an aspect not previously pursued in the original research, which primarily focused on the molecular landscape of the over 183k cells in human brain hippocampus vasculature in AD^31^. Commencing with a UMAP visualization of the single-cell data, color-coded by cell type (Figure 5A), we achieved a broad perspective of 13 cell types (i.e., Arterial cell, Astrocyte, Capillary cell, Ependymal cell, Fibroblast, Microglia, Neuron, Oligodendrocyte progenitor cell, Oligodendrocytes, Pericyte, Smooth muscle cell, T cell, and Venous). From the ensuing breakdown of the proportion and count of each cell type by sex (Figure 5B–C), we observe more Oligodendrocytes, Astrocytes, and Microglia in overall female cell types than male. Such sex-specific differences in cellular composition raise intriguing questions about the potential roles of these variations in disease pathogenesis and progression, warranting further investigation. We further compared DEGs across four demographic groups: Male AD patients, Female AD patients, Male controls, and Female controls. The result showcased both unique and shared gene signatures among these groups (Figure 5D). Our findings indicate that both sex and disease status can shape the transcriptomic landscape of cells, which could have profound implications for understanding the underlying molecular mechanisms of AD and developing targeted therapies.

Examining expression profiles of ten selected genes across five cell types (i.e., Microglia, Oligodendrocytes, Pericytes, Astrocytes, and Fibroblasts), segregated by sex, revealed unique sex-specific gene expression patterns within different cell types (Figure 5E). For example, the myocyte-specific enhancer factor 2A (MEF2A) is upregulated in Microglia in Female to establish microglial-specific enhancer profiles^32^. Our analysis of Gene Set Enrichment Analysis (GSEA) further explored the activity of binding and uptake of ligands by scavenger receptors, which are known to be involved in the recognition and clearance of various ligands, including modified lipoproteins, cellular debris, and pathogens^33^. In AD, the dysregulation of scavenger receptor activity has been implicated in the clearance of Aβ plaques, which are one of the hallmark pathological features of the disease^34^. Previous studies have reported sex differences in scavenger receptor expression in the context of AD^35^. Additionally, we observed differences in GSEA enrichment of neurogenesis pathways in Microglia under AD conditions. Microglia can shape adult hippocampal neurogenesis^36^. We compared Male AD vs. Male Control (Figure 5G), Female AD vs. Female Control (Figure 5H), and Male AD vs. Female AD (Figure 5I). Interestingly, neurogenesis pathway activity varied significantly, with upregulation observed in female AD, and downregulation in male AD patients. This observation denotes a sex-dependent dysregulation of neurogenesis in AD, indicating that the disease could affect foundational neural processes in a sex-specific manner. Enhancing our understanding of how microglial activation states differentially regulate adult neurogenesis in men and women could yield invaluable insights into the disease’s intricate mechanisms. Taken together, these results underscore the intricate interplay between sex and cellular and molecular profiles in AD. They highlight the necessity of considering sex as an integral factor in AD research, and point towards the potential for developing more personalized, sex-specific therapeutic strategies in the future.

## Discussion

We develop ssREAD, a single-cell and spatial RNA-seq database for AD, not only accommodates the expansion of sc/snRNA-seq data but is uniquely poised to incorporate and harness the emergent wealth of AD-related ST data. ssREAD houses data encompassing various species, diseases, tissues, and cell types, thereby, permitting granular analyses that could reveal intricate biological phenomena. ssREAD’s capabilities are broad, allowing for diverse analytical activities such as contrasting diseased tissues against healthy controls, identifying distinct cell types through gene markers, and examining gene expression across diverse tissues and cell types. The most compelling findings of sex differences in AD unveil a profound heterogeneity between male and female cellular profiles. Particularly, females exhibited a heightened presence of Oligodendrocytes, Astrocytes, and Microglia compared to males. Furthermore, while female AD patients showed upregulation in neurogenesis pathway activity, their male counterparts displayed a stark downregulation. These insights align with the findings of previous studies claiming that transcriptional responses were substantially different between sexes in different cell types^37^, underscoring the pivotal role of gender in shaping AD’s cellular and molecular features. Last but not least, designed with the end-user in mind, ssREAD is characterized by its user-friendly interface, query function, and visualizations. In terms of its infrastructure, ssREAD utilizes high-performance computing to manage large-scale single-cell data analysis efficiently. It comprises a cutting-edge server architecture employing diverse programming languages, machine learning frameworks, and data visualization libraries. As our understanding of the complexity and heterogeneity of biological systems continues to deepen, tools like ssREAD can play an increasingly vital role in our pursuit and understanding of AD studies.

To widen the accessibility and integration of ssREAD, we intend to develop an R package within the Bioconductor project and a Python library in the future, enabling users to access all datasets both locally and remotely through ssREAD’s server-side API. Future plans also include expanding our analytical pipelines and visualization methodologies to enrich the capabilities of ssREAD. More AD datasets will be collected and incorporated with ssREAD. Recognizing the need for a platform that accommodates the diverse range of interests in the scientific community, we plan to include a wider range of sc/snRNA-seq and spatial omics data of neural systems such as human iPSC-derived neurons, glia, and neural organoids. Furthermore, we aspire to include more neurodegenerative diseases like Frontotemporal lobar degeneration, Parkinson’s disease, and Amyotrophic lateral sclerosis, serving a broader research community.

## Methods

### ST Data Preprocessing

Spatial transcriptomics data preprocessing was performed using the Seurat and Squidpy packages. Seurat was employed for quality control, normalization, and identification of highly variable genes^38^. Squidpy was used for spatially-resolved computations, such as spatial autocorrelation analysis, extracting neighborhood information, and spatially-resolved clustering^39^.

### Spatially Variable Genes (SVGs) identification

To identify spatially variable genes, the spaGCN tool was utilized^11^. It integrates graph convolutional network (GCN) with spatial transcriptomics to detect genes that vary significantly across different spatial locations in tissue samples.

### Cell type annotation

We applied a similar cell type annotation strategy used in scREAD. Our quest for analytical precision led us to modify our initial cell type annotation strategy to increase annotation accuracy. We recognized that several marker genes used in the original release demonstrated sub-optimal specificity. To address this, we executed two iterations of cell type annotation, which involved filtering out several less specific markers. During the first iteration, only Neurons were annotated. Following this, the Neurons were then isolated from the complete dataset, and second iteration marker genes were employed to annotate Excitatory neurons and Inhibitory neurons. The cell labels for Neurons were subsequently replaced by those of Excitatory neurons or Inhibitory neurons. This revised workflow enhances the annotation quality by accounting for the discrepancies between different cell types and subtypes.

We used SCINA R package that leverages prior marker genes information and simultaneously performs cell type clustering and assignment for known cell types^40^. Furthermore, SCINA shows good performances among prior-knowledge classifiers when high-quality marker genes are provided^41^. Each cell was assigned a cell type based on a manually created marker gene list file using SCINA v1.2.0, whereas the cells with unknown labels marked by SCINA were first compared with predicted clusters from Seurat, and then the unknown labels were assigned to the most dominant cell types within the predicted clusters.

### Cell Type Deconvolution Analysis

Deconvolution of mixed cell populations was performed using the CARD R package^30^, which employs a reference-based approach to estimate the proportions of different cell types in bulk RNA-seq or spatial transcriptomics data. It uses cell-type-specific gene expression signatures from a reference dataset to calculate cell type proportions.

### Differential Gene Expression Analysis

The differential gene expression analysis was performed using the non-parametric Wilcoxon rank sum test in Seurat’s FindMarkers function. This function identifies genes that are differentially expressed between different conditions, such as disease versus control, or male versus female. For each comparison, the computed p-values were adjusted to control the false discovery rate (FDR) using the Bonferroni correction for all genes in the dataset. Genes with FDR <0.05 were considered as differentially expressed genes. Over-representation enrichment analysis was performed to identify gene ontology terms using the Enrichr R package (v.3.0) on the Reactome pathway database^42,43^. The p-value from Enrichr was computed using a hypergeometric test which is a binomial proportion test that assumes a binomial distribution and independence for the probability of any gene belonging to any set. The q-value from Enrichr is an adjusted p-value using the Benjamini-Hochberg method for correction for multiple hypotheses testing. Enrichr precomputes a background expected rank for each term in each gene set library, which is a lookup table of expected ranks and variances for each term in the library. These expected values are precomputed using the hypergeometric test for many random input gene sets for each term in the gene set library.

### Tissue Architecture Identification

The tissue architecture was analyzed using RESEPT^13^, a computational tool that generates an atlas of regional gene expression patterns in spatial transcriptomics data. This tool enables the visualization of how gene expression varies across different spatial regions within a tissue sample.

### Gene Regulatory Network Analysis

Regulatory analysis was conducted using DeepMAPS^44,45^. We employed DeepMAPS to build the cell and gene embeddings and obtained active gene modules through the graph transformer model. The gene modules were further sent to DeepMAPS to perform cell cluster active gene module determination, de novo motif finding, and TF matching and CTSR determination with the default parameters. Gene regulatory networks were constructed to indicate the predicted TF-gene regulatory relations via Cytoscape^46,47^.

### User interface

We made substantial improvements to the user interface to enhance accessibility and ease of use. Key modifications were made within the dataset details page, such as the addition of gene expression display for single cells/spots using feature plots and violin plots. We also introduced a dedicated query page for DEG searching. Notably, search results can now be refined based on sex, group, and condition parameters. The new design offers clearer demarcation between DEG searching and query options. Moreover, we integrated the function to calculate overlapping DEGs from multiple comparisons, an approach originally outlined in the scREAD protocol^48^. This functionality is now delivered through an interactive online query. We also redesigned the homepage for better usability, positioning the search bar at the top and displaying key information about the number of species, studies, assays, and versions for higher visibility.

### ssREAD server construction

ssREAD is hosted on an HPE XL675d RHEL system outfitted with a 2×128-core AMD EPYC 7H12 CPU, 64GB RAM, and 8×NVIDIA A100 80GB GPU. Our backend server, written in TypeScript and built with the koa.js framework, leverages Auth0 to provide independent user authentication and authorization services. Our frontend is constructed with NUXT, utilizing Vuetify as the UI library and Plotly.js for data visualization. Communication between frontend and backend servers is enabled using REST API. This streamlined server construction ensures robust, efficient, and scalable performance of ssREAD.

## Data Availability

All datasets used in this work are available from publicly available sources as cited in the manuscript. ssREAD is a one-stop and user-friendly interface and is freely available at https://bmblx.bmi.osumc.edu/ssread/. The frontend code is available at https://github.com/OSU-BMBL/ssread. The backend code is available at https://github.com/OSU-BMBL/ssread-backend.
